# Supplementary material for: Evaluating the Effectiveness of Various Small RNA Alignment Techniques in Transcriptomic Analysis by Examining Different Sources of Variability Through a Multi-Alignment Approach
Source: Methods Protoc. 2025 Jun 17;8(3):65. doi: 10.3390/mps8030065 (PMC12195907; doi:10.3390/mps8030065)
Supplement: Supplementary file 1 [file mps-08-00065-s001.zip › Figure S1.pdf]

Figure S1. Venn diagrams of the differential sets in DE-1 and DE-2.

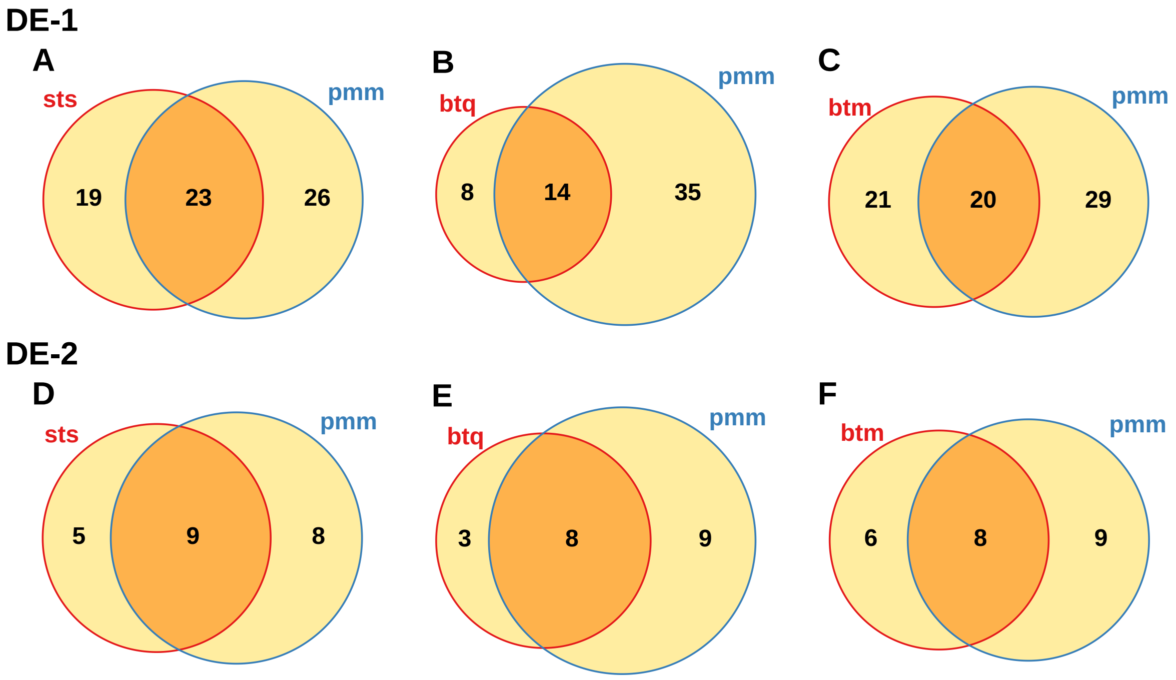

Panel 'DE-1' is showing in A to C the overlap between the three different alignment and quantification approaches and their significant microRNA candidates for the first differential analysis. The nomenclature is already described in Table 2 in the right column.
In panel 'DE-2' the same is done in D to E for the second differential analysis and again on microRNAs.
The two differential analyses differentiate by absolute count number and a more pronounced higher set number for the premature microRNAs (pmm).
Acronyms sts, btq, btm, pmm explainded in supplementary Table S1.
