# Supplementary material for: Evaluating the Effectiveness of Various Small RNA Alignment Techniques in Transcriptomic Analysis by Examining Different Sources of Variability Through a Multi-Alignment Approach
Source: Methods Protoc. 2025 Jun 17;8(3):65. doi: 10.3390/mps8030065 (PMC12195907; doi:10.3390/mps8030065)
Supplement: Supplementary file 1 [file mps-08-00065-s001.zip › Table S1.pdf]

Table S1. Overview on resources, files and terms.

All references here are based on Homo sapiens.

**Program for alignment**	**STAR**	**STAR**	**STAR**	**Bowtie2**	**Bowtie2**	**Bowtie2**	**BBMap**
Fasta file	mt.trans.hu.miRtRNA.<br>111.primary.fa	Homo_sapiens.<br>GRCh38.111.dna.<br>primary_assembly.fa	mte.trans.hu.GRCh38.<br>111.primary.fa	m2_miRtRNA.fasta	Homo_sapiens.<br>GRCh38.111.dna.<br>primary_assembly.fa	r_star_pirbase_gold<br>.fasta	m2_miRtRNA.fasta
Index folder	star2711x111HUmt	star2711x111HUpmm	star2711x111HUmte	bt2_mt	bt2_111HU	bt2_pir	bb_mt
GTF file	mt.Homo_sapiens.<br>miRtRNA.gtf	Homo_sapiens.<br>GRCh38.111.<br>mirbase.gtf	mte.Homo_sapiens.<br>GRCh38.111.gtf	no gtf	mt.Homo_sapiens.<br>miRtRNA.gtf<br>(for QualiMap)	no gtf	no gtf
Program parameter	outFilterMismatchNmax 0,<br>outFilterMultimapNmax 2,<br>outSAMmultNmax 1,<br>quantMode<br>TranscriptomeSAM.	outFilterMismatchNmax 0,<br>outFilterMultimapNmax 2,<br>outSAMmultNmax 1,<br>quantMode<br>TranscriptomeSAM.	outFilterMismatchNmax 0,<br>outFilterMultimapNmax 2,<br>outSAMmultNmax 1,<br>quantMode<br>TranscriptomeSAM.	--end-to-end	--local	--local	no special features used<br>(global)
Program to quantify	a) Salmon<br>b) Samtools idxstats	a) Samtools idxstats	a) Salmon<br>b) Samtools idxstats	a) Samtools idxstats	a) Samtools idxstats **<br>b) Qualimap count	a) Samtools idxstats	a) Samtools idxstats
Result acronyms	a) sts<br>b) stm	a) pmm	a) **<br>b) mte **	a) btm	a) chr **<br>b) btq	a) pi	a) bbm
Result folder name	03star1out	03star2out	03star3out	04bt21out	04bt22out	04bt23out	05bbm1out
Quantification output file(s)	a) quant.sf<br>b) mcounts.tsv	a) mcounts.tsv	a) mcounts.tsv	a) mcounts.tsv	a) mcounts.tsv **<br>b) qm.111HUmte.tsv	a) mcounts.tsv	a) mcounts.tsv
Reference features	2653 mirbase microRNAs,<br>260 tRNAs	1852 premature<br>microRNAs	39 gene_biotype items<br>with now 4114450 (v113)<br>elements	2653 mirbase microRNAs,<br>260 tRNAs	39 gene_biotype items<br>with now 4114450 (v113)<br>elements	19222 pirbase small RNAs	2653 mirbase microRNAs,<br>260 tRNAs
Result id type	ENST - extra ENST<br>names for micro/tRNAs<br>(will be translated by R<br>functions to a microRNA<br>[mirbase] or tRNA name<br>[tRNA database])	regular ENST name of<br>premature microRNAs	regular ENST name of all<br>features included in<br>standard Homo_sapiens.<br>GRCh38.111	mirbase name	chromosome and contig<br>names<br>respectively<br>ENSG micro/tRNA name	pirbase name	mirbase name
Script function name	**staroutA**	**staroutB**	**staroutC**	**bt2outA**	**bt2outB**	**bt2outC**	**bbmoutA**
Support file for R import	namelist.txt	namelist.txt	namelist.txt	namelist.txt	namelist.txt	namelist.txt	namelist.txt

Note :

1) Not every aligner is used in all three script files.

2) ** : not used here.
