# Supplementary material for: Evaluating the Effectiveness of Various Small RNA Alignment Techniques in Transcriptomic Analysis by Examining Different Sources of Variability Through a Multi-Alignment Approach
Source: Methods Protoc. 2025 Jun 17;8(3):65. doi: 10.3390/mps8030065 (PMC12195907; doi:10.3390/mps8030065)
Supplement: Supplementary file 1 [file mps-08-00065-s001.zip › Table S2.pdf]

Table S2. View on the replication of significant candidates between the different approaches.

Only two replicating candidates are shown in blue to exemplify the concept of the table.
Cf. also Figure 5.
Table sorted top to bottom by increasing p value.

DE-1

STAR mt index, Salmon quantification	STAR mt index, Samtools quantification	Bowtie2 mt index, Samtools quantification	Bowtie2 Qualimap quantification, mt reference	BBMap mt index, Samtools quantification	STAR pmm index, Samtools quantification
sts	stm	btq	btm	bbm	pmm
hsa-miR-106b-3p	hsa-miR-106b-3p	hsa-miR-425-5p	hsa-miR-106b-3p		MIR106B
hsa-miR-425-5p	hsa-miR-425-5p	hsa-miR-200a-3p	hsa-miR-425-5p		MIR31
hsa-miR-363-3p	hsa-miR-363-3p	hsa-miR-136-3p	hsa-miR-532-5p		MIR532
hsa-miR-532-5p	hsa-miR-532-5p	hsa-miR-25-3p	hsa-miR-363-3p		MIR210
hsa-miR-210-3p	hsa-miR-210-3p	hsa-miR-363-3p	hsa-miR-199a-3p		MIR30B
hsa-let-7d-3p	hsa-let-7d-3p	hsa-miR-16-5p	hsa-miR-210-3p		MIR363
hsa-miR-181a-2-3p	hsa-miR-181a-2-3p	hsa-miR-181a-2-3p	hsa-miR-221-3p		MIR185
hsa-miR-185-5p	hsa-miR-185-5p	hsa-miR-132-3p	hsa-miR-185-5p		MIR497
hsa-miR-132-3p	hsa-miR-221-3p	hsa-miR-221-3p	hsa-miR-17-3p		MIR30C2
hsa-miR-221-3p	hsa-miR-132-3p	hsa-miR-199a-3p	hsa-let-7d-3p		MIR30C1
hsa-miR-25-3p	hsa-miR-25-3p	hsa-miR-532-5p	hsa-miR-103a-3p		MIR193B
hsa-miR-30c-5p	hsa-miR-30c-5p	hsa-miR-185-5p	hsa-miR-25-3p		MIR34C
hsa-miR-484	hsa-miR-484	hsa-let-7d-3p	hsa-miR-199b-3p		MIR1307
hsa-miR-17-3p	hsa-miR-17-3p	hsa-miR-484	hsa-miR-30c-5p		MIR181A2
hsa-miR-34c-5p	hsa-miR-34c-5p	hsa-miR-30b-5p	hsa-miR-132-3p		MIR25
hsa-miR-30b-5p	hsa-miR-30b-5p	hsa-miR-93-5p	hsa-miR-1307-3p		MIR16-2
hsa-miR-106b-5p	hsa-miR-106b-5p	hsa-miR-361-3p	hsa-miR-106b-5p		MIR17
hsa-miR-1307-3p	hsa-miR-1307-3p	hsa-miR-181a-5p	hsa-miR-34c-5p		MIR16-1
hsa-miR-21-3p	hsa-miR-21-3p	hsa-miR-199b-5p	hsa-miR-424-5p		MIR652
hsa-miR-135a-5p	hsa-miR-135a-5p	hsa-miR-135a-5p	hsa-miR-181a-2-3p		MIR181A1
hsa-miR-128-3p	hsa-miR-128-3p	hsa-miR-34c-5p	hsa-miR-95-3p		MIR382
hsa-miR-16-5p	hsa-miR-16-5p	hsa-miR-22-3p	hsa-miR-21-3p		MIR23A
hsa-miR-21-5p	hsa-miR-21-5p		hsa-miR-128-3p		MIR128-1
hsa-miR-652-3p	hsa-miR-652-3p		hsa-miR-484		MIR425
hsa-miR-455-5p	hsa-miR-455-5p		hsa-miR-140-5p		MIR93
hsa-miR-181a-5p	hsa-miR-181a-5p		hsa-miR-7-5p		MIR199B
hsa-miR-140-5p	hsa-miR-140-5p		hsa-miR-1275		MIR660
hsa-miR-199b-5p	hsa-miR-199b-5p		hsa-miR-21-5p		MIR135A2
hsa-miR-424-5p	hsa-miR-424-5p		hsa-miR-30a-3p		MIR128-2
hsa-miR-3184-5p	hsa-miR-3184-5p		hsa-miR-652-3p		MIR451A
hsa-miR-31-5p	hsa-miR-31-5p		hsa-miR-16-5p		MIR451B
hsa-miR-95-3p	hsa-miR-95-3p		hsa-miR-107		MIR1287
hsa-miR-423-3p	hsa-miR-423-3p		hsa-miR-30b-5p		MIR21
hsa-miR-181a-3p	hsa-miR-181a-3p		hsa-miR-135a-5p		MIR22
hsa-miR-337-3p	hsa-miR-337-3p		hsa-miR-486-3p		MIR103A1
hsa-miR-144-3p	hsa-miR-144-3p		hsa-miR-409-3p		MIR26B
hsa-miR-361-3p	hsa-miR-361-3p		hsa-miR-181a-5p		MIR103A2
hsa-miR-30a-3p	hsa-miR-30a-3p		hsa-miR-337-3p		MIR629
hsa-miR-22-3p	hsa-miR-22-3p		hsa-miR-574-3p		MIR181D
hsa-miR-26b-5p	hsa-miR-103a-3p		hsa-miR-30b-3p		MIR103B1
hsa-miR-200a-5p	hsa-miR-103b		hsa-miR-629-5p		MIR103B2
hsa-miR-30b-3p	hsa-miR-26b-5p		hsa-miR-103b		MIR95
hsa-miR-103b	hsa-miR-200a-5p		hsa-miR-199b-5p		MIR337
hsa-miR-103a-3p	hsa-miR-30b-3p		hsa-miR-455-5p		MIR132
hsa-miR-486-3p	hsa-miR-486-3p		hsa-miR-361-3p		MIR92A1
hsa-miR-23a-5p	hsa-miR-23a-5p				MIR484
					MIR339
					MIR29A
					MIR136

DE-2

STAR mt index, Salmon quantification	STAR mt index, Samtools quantification	Bowtie2 mt index, Samtools quantification	Bowtie2 Qualimap quantification, mt reference	BBMap mt index, Samtools quantification	STAR pmm index, Samtools quantification
sts	stm	btq	btm	bbm	pmm
hsa-miR-193b-5p	hsa-miR-193b-5p	hsa-miR-193b-5p	hsa-miR-193b-5p	hsa-miR-4668-5p	MIR320A
hsa-miR-320a-3p	hsa-miR-320a-3p	hsa-miR-320a-3p	hsa-miR-320a-3p	hsa-miR-193b-5p	MIR193B
hsa-miR-483-5p	hsa-miR-483-5p	hsa-miR-483-5p	hsa-miR-483-5p		MIRLET7B
hsa-miR-432-5p	hsa-miR-432-5p	hsa-miR-432-5p	hsa-let-7b-5p		MIR432
hsa-let-7b-5p	hsa-let-7b-5p	hsa-miR-664a-5p	hsa-miR-4454		MIR320B2
hsa-miR-320b	hsa-miR-320b	hsa-miR-335-5p	hsa-miR-432-5p		MIR320B1
hsa-miR-561-5p	hsa-miR-664a-5p	hsa-let-7b-5p	hsa-miR-664a-5p		MIR561
hsa-miR-664a-5p	hsa-miR-433-3p	hsa-miR-561-5p	hsa-miR-433-3p		MIR19B1
hsa-miR-1307-5p	hsa-miR-19b-3p	hsa-miR-1180-3p	hsa-miR-12136		MIR191
hsa-miR-433-3p	hsa-miR-191-5p	hsa-miR-342-3p	hsa-miR-191-5p		MIR671
hsa-miR-19b-3p	hsa-miR-342-3p	hsa-miR-433-3p	hsa-miR-342-3p		MIR19B2
hsa-miR-191-5p	hsa-miR-379-5p		hsa-miR-1260b		MIR2110
hsa-miR-342-3p			hsa-miR-379-5p		MIR664A
hsa-miR-379-5p			hsa-miR-1260a		MIR379
					MIR4521
					MIR1180
					MIR433
