# Supplementary material for: Evaluating the Effectiveness of Various Small RNA Alignment Techniques in Transcriptomic Analysis by Examining Different Sources of Variability Through a Multi-Alignment Approach
Source: Methods Protoc. 2025 Jun 17;8(3):65. doi: 10.3390/mps8030065 (PMC12195907; doi:10.3390/mps8030065)
Supplement: Supplementary file 1 [file mps-08-00065-s001.zip › Table S3.pdf]

Table S3. Looking into the real counts of two selected microRNAs discussed in the ‘Method Validation’ section.

DE-2

bbm	baseMean	log2FC	lfcSE	stat	pvalue	padj	EH01	EH02	EH03	EH04	EH05	EH06	EH07	EH08	EH09	EH10	EH11	EH12	EH13	EH14	EH15	EH16	EH17	E01	E02	E03	E04	E06	E07	E08	E09	E10	E11	E12	E13	E14	E15
hsa-miR-193b-5p	3.484	-1.606	0.361	-4.451	8.54E-06	8.60E-03	1	3	4	4	4	3	1	6	7	7	3	2	5	4	5	6	3	3	2	0	0	0	0	0	1	0	1	1	0	0	1
hsa-miR-4668-5p	5.387	-1.635	0.297	-5.502	3.75E-08	7.55E-05	6	8	8	3	9	4	6	6	9	12	6	5	6	3	7	6	11	3	1	1	5	0	0	4	0	1	1	4	1	0	0

sts	baseMean	log2FC	lfcSE	stat	pvalue	padj	EH01	EH02	EH03	EH04	EH05	EH06	EH07	EH08	EH09	EH10	EH11	EH12	EH13	EH14	EH15	EH16	EH17	E01	E02	E03	E04	E06	E07	E08	E09	E10	E11	E12	E13	E14	E15
hsa-miR-193b-5p	6.839	-2.319	0.365	-6.349	2.16E-10	7.05E-08	4	15	12	6	9	3	4	11	11	16	9	11	12	3	7	21	11	7	0	0	2	0	1	0	0	0	2	1	3	0	0
hsa-miR-4668-5p	1.001	0.002	0.566	0.004	9.97E-01	1.00E+00	0	0	0	0	0	0	0	0	0	0	0	0	0	0	0	0	0	0	0	0	0	0	0	0	0	0	0	0	0	0	0

stm	baseMean	log2FC	lfcSE	stat	pvalue	padj	EH01	EH02	EH03	EH04	EH05	EH06	EH07	EH08	EH09	EH10	EH11	EH12	EH13	EH14	EH15	EH16	EH17	E01	E02	E03	E04	E06	E07	E08	E09	E10	E11	E12	E13	E14	E15
hsa-miR-193b-5p	6.839	-2.319	0.365	-6.361	2.01E-10	5.73E-08	4	15	12	6	9	3	4	11	11	16	9	11	12	3	7	21	11	7	0	0	2	0	1	0	0	0	2	1	3	0	0
hsa-miR-4668-5p	1.001	0.002	0.562	0.004	9.97E-01	1.00E+00	0	0	0	0	0	0	0	0	0	0	0	0	0	0	0	0	0	0	0	0	0	0	0	0	0	0	0	0	0	0	0

btq	baseMean	log2FC	lfcSE	stat	pvalue	padj	EH01	EH02	EH03	EH04	EH05	EH06	EH07	EH08	EH09	EH10	EH11	EH12	EH13	EH14	EH15	EH16	EH17	E01	E02	E03	E04	E06	E07	E08	E09	E10	E11	E12	E13	E14	E15
hsa-miR-193b-5p	5.162	-2.292	0.385	-5.954	2.62E-09	7.83E-07	3	12	9	2	7	2	4	9	6	11	6	10	8	1	5	15	10	2	0	0	2	0	1	0	0	0	1	0	3	0	0
hsa-miR-4668-5p	1.001	0.002	0.564	0.004	9.97E-01	1.00E+00	0	0	0	0	0	0	0	0	0	0	0	0	0	0	0	0	0	0	0	0	0	0	0	0	0	0	0	0	0	0	0

btm	baseMean	log2FC	lfcSE	stat	pvalue	padj	EH01	EH02	EH03	EH04	EH05	EH06	EH07	EH08	EH09	EH10	EH11	EH12	EH13	EH14	EH15	EH16	EH17	E01	E02	E03	E04	E06	E07	E08	E09	E10	E11	E12	E13	E14	E15
hsa-miR-193b-5p	9.258	-2.072	0.325	-6.379	1.79E-10	4.98E-08	6	18	13	8	10	11	5	13	16	24	11	13	16	5	9	28	17	8	2	0	4	0	2	1	0	3	5	2	4	0	2
hsa-miR-4668-5p	1.000	0.000	0.537	0.000	1.00E+00	1.00E+00	0	0	0	0	0	0	0	0	0	0	0	0	0	0	0	0	0	0	0	0	0	0	0	0	0	0	0	0	0	0	0

Mirbase

```
Accession      MIMAT0004767
Description    hsa-miR-193b-5p mature miRNA

Sequence

14 - CGGGGUUUUGAGGGCGAGAUGA - 35

Hairpins

            u              G  A  A  uua
guggucucagaa CGGGGUUUUGAGGGC AG UG gu   u
|||||||||||| ||||||||||||||| || || ||   g
uacugggguuuU GCCCUGAAACUCCCG UC Ac ua   u
            C              G  A  c  uuu

guggucucagaauCGGGGUUUUGAGGGCGAGAUGAguuuauguuuauccAACUGGCCCUCAAAGUCCCGCUuuuggggucau
((((((((((((.(((((((((((((((.((.((.((.........)).)).)).))))))))))))))).))))))))))))
```

```
Accession      MIMAT0019745
Description    hsa-miR-4668-5p mature miRNA

Sequence

1 - AGGGAAAAAAAAAAAGGAUUUGUC - 23

Hairpin

-A       A           G    gu  c   g
  GGGAAAA AAAAAGGAUUU UCuu  ag cag a
  ||||||| ||||||||||| ||||  || |||
  CCUUUUU UUUUUCCUAAA AGaa  uu guu u
GA       G           -    au  u   a

AGGGAAAAAAAAAAAGGAUUUGUCuuguagccaggauauuguuuuaaaGAAAAUCCUUUUUGUUUUUCCAG
.(((((((.(((((((((((.((((..((.(((....))).))..)))))))))))))))).)))))))..
```

Distribution of reads

Sample number : 85

Name	Molecule counts	Negative samples	Range per sample
miR-193b-5p	410	9	0-28
miR-193b-3p	339	18	0-63
miR-4668-5p	3	83	0-2
miR-4668-3p	0	85	NA

Motif per read

Name	Dominant motif	Very rare
miR-193b-5p	CGGGGTTTTGAGGGCGAGATGA	TTTGAGGGCGAGATGA<br>CGGGGTTTTGAGGGCGAGAT
miR-193b-3p	AACTGGCCCTCAAAGTCCCGCT	ACTGGCCCTCAAAGTCCCGCT<br>AACTGGCCCTCAAAGTCCCGC
miR-4668-5p	AGGGAAAAAAAAAAAGGATTTGT	/
miR-4668-3p	/	/
